# Biochemical Changes and Molecular Mechanisms Mediated by Sulfur Dioxide in Healthy Skin and Dermatological Disorders

**DOI:** 10.3390/biom16060915

**Published:** 2026-06-19

**Authors:** Mircea Tampa, Ilinca Nicolae, Madalina Irina Mitran, Cristina Iulia Mitran, Clara Matei, Milena Tocut, Simona Roxana Georgescu, Cosmin Ene, Cristina Capusa, Corina Daniela Ene

**Affiliations:** 1 Department of Dermatology, ‘Carol Davila’ University of Medicine and Pharmacy, 020021 Bucharest, Romania; mircea.tampa@umfcd.ro; 2 Department of Dermatology, ‘Victor Babes’ Clinical Hospital for Infectious Diseases, 030303 Bucharest, Romania; drnicolaei@yahoo.ro; 3 Department of Microbiology, ‘Carol Davila’ University of Medicine and Pharmacy, 020021 Bucharest, Romania; irina.mitran@umfcd.ro (M.I.M.); cristina.mitran@umfcd.ro (C.I.M.); 4 Israel Faculty of Medicine, Tel Aviv 69978, Israel; milena.tocut@gmail.com; 5 Internal Medicine C Department, Wolfson Medical Center, Holon 58100, Israel; 6 Department of Urology, ‘Carol Davila’ University of Medicine and Pharmacy, 020021 Bucharest, Romania; cosmin.ene@umfcd.ro; 7 Department of Urology, “Saint John” Emergency Clinical Hospital, 042122 Bucharest, Romania; 8 Department of Nephrology, ‘Carol Davila’ University of Medicine and Pharmacy, 020021 Bucharest, Romania; cristina.capusa@umfcd.ro (C.C.); corina.ene@umfcd.ro (C.D.E.); 9 Department of Nephrology, ‘Carol Davila’ Nephrology Hospital, 010731 Bucharest, Romania

**Keywords:** sulfur dioxide, toxic agent, biological modulator, skin physiology, skin disorders

## Abstract

The skin serves as the body’s first line of defense against environmental threats, acting as a barrier between external aggressors and internal systems. Current evidence regarding the roles of sulfur dioxide (SO_2_) in biology and medicine is limited. Environmental pollutants, including SO_2_, can increase the production of reactive oxygen species in the skin, leading to oxidative damage that may worsen various dermatological conditions. Endogenous SO_2_, proposed as the fourth member of the gasotransmitter family, functions as a biological signaling molecule. It is generated in various human skin cells, including vascular smooth muscle cells, endothelial cells, mast cells, keratinocytes, macrophages, adipocytes, fibroblasts, dermal immune cell population, etc, where it performs multiple functions at physiologically relevant concentrations. Endogenous SO_2_ plays a crucial role in regulating cell signaling and maintaining skin homeostasis through its antioxidant, anti-inflammatory, and cytoprotective effects. Abnormal generation and metabolism of SO_2_ are linked to several critical processes in the skin, including vascular biology, immune response, cell proliferation, pigmentation, malignancy, protective barriers, senescence, and resistance to stress. This paper provides a narrative review of the significant roles of SO_2_ in skin health and disease. A comprehensive understanding of the complex molecular effects and mechanisms mediated by SO_2_ in human skin, along with the development of gas therapy, will be essential for translating fundamental research into clinical applications.

## 1. Introduction

The skin is the largest organ of the body, divided into three main layers: the epidermis, the dermis, and the hypodermis. The primary skin cells include keratinocytes (which produce keratin that results after the terminal differentiation of keratinocytes and help renew the skin barrier), dendritic cells, fibroblasts (located in the dermis and responsible for producing collagen, elastin, and hyaluronic acid), mast cells, macrophages, and adipocytes [1]. Human skin plays a crucial role in protecting the body against various stressors, but it is particularly susceptible to oxidative damage [2].

Recent research highlights the role of sulfur dioxide (SO_2_) as both an external pollutant (a toxic agent) and a gasotransmitter (a modulating agent involved in physiological and pathological processes in the skin) [3,4]. Studies have identified a significant correlation between both short-term and long-term exposure to air pollutants and the exacerbation of several chronic dermatological conditions, including atopic dermatitis, psoriasis, urticaria, acne [5,6]. The skin’s response to environmental stressors reveals a complex relationship among barrier function, immune surveillance, and structural integrity. The mechanisms connecting external pollution to skin pathology involve excessive generation of reactive oxygen species, pro-inflammatory signaling, skin barrier dysfunction, increased transepidermal water loss, and microbiome dysbiosis [5].

Recently, the regulatory roles of gasotransmitters in skin physiology and pathology have become more evident [7,8]. Gasotransmitters play essential roles in cell signaling and maintaining skin homeostasis. The interactions among nitric oxide (NO), hydrogen sulfide (H_2_S), carbon monoxide (CO), and sulfur dioxide (SO_2_) in the skin constitute a finely regulated network that significantly influences both physiological and pathological processes. Although each gasotransmitter possesses distinct biosynthetic pathways and primary molecular targets, growing evidence indicates that they operate as an integrated signaling system that finely modulates essential cellular functions [7].

Endogenous SO_2_ is recognized for its potent antioxidant, anti-inflammatory, and cytoprotective properties [7,9]. SO_2_ has been shown to neutralize oxygen free radicals, improve mitochondrial function, and reduce cellular senescence, making it a promising candidate for combating oxidative stress. An important characteristic of SO_2_ is its dual nature; it exerts a modulatory function at low, controlled concentrations but can become toxic when levels exceed physiological thresholds [6,10].

The purpose of this review is to examine the primary roles that SO_2_ plays in the physiological and pathophysiological processes of the skin, as well as the potential efficacy of gas therapy in treating dermatological conditions. The following sections will present the current research directions and advancements regarding: the characteristics of SO_2_ as a member of the gasotransmitter family, its role in maintaining skin homeostasis, the potential relationship between SO_2_ dynamics and skin pathology, and the interactions between SO_2_ and key skin cells (keratinocytes, melanocytes, fibroblasts, mast cells, vascular cells, macrophages), as well as the skin microbiome, along with associated molecular mechanisms. Investigating the relationship between SO_2_ and skin health is crucial for advancing our understanding of SO_2_ as both a biological modulator and a potential therapeutic agent, which could lead to new treatment options for chronic skin diseases and help mitigate cellular aging processes.

## 2. The Metabolism and Signaling of Endogenous SO_2_ in Healthy Skin and Dermatological Diseases

**Figure 1:**
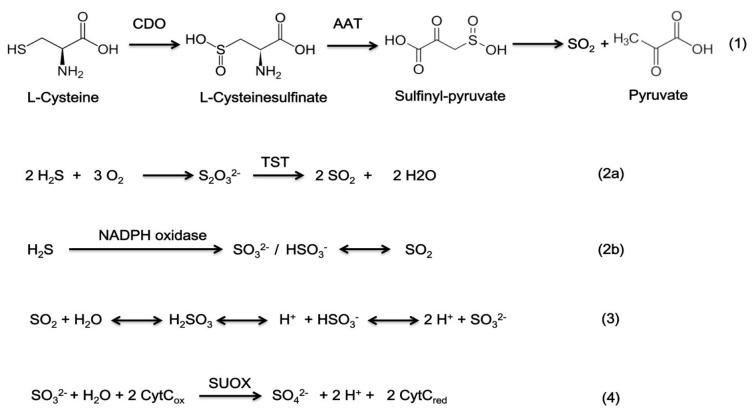
Endogenous synthesis of SO_2_. CDO—cysteine dioxygenase; AAT—aspartate aminotransferase; TST—thiosulfate sulfide transferase; SUOX—sulfite oxidase; SO_2_—sulfur dioxide; CytCox—oxidised cytochrome; CytCred—reduced cytochrome c.

Under physiological conditions, endogenous SO_2_ is synthesized in human skin cells—including vascular smooth muscle cells, endothelial cells, fibroblasts, dermal immune cell population, mast cells, keratinocytes, macrophages, and adipocytes—through enzymatic pathways that produce precise quantities at appropriate times. The primary pathway for endogenous SO_2_ generation involves the conversion of sulfur amino acids (cysteine and methionine) to L-cysteine sulfinate (a reaction catalysed by cysteine dioxygenase—CDO), followed by its transformation into sulfinylpyruvate through the action of aspartate aminotransferase (AAT) (Figure 1). Ultimately, this process leads to the production of pyruvate and SO_2_ through spontaneous decomposition (Reaction 1). Another route for SO_2_ synthesis occurs when H_2_S is converted to sulfite or SO_2_ by the action of NADPH oxidase in activated neutrophils. Additionally, H_2_S can be oxidized in vivo to thiosulfate, which is then converted to SO_2_ under the action of thiosulfate sulfide transferase (TST) (Reaction 2). In the body, SO_2_ is rapidly hydrated into sulfites and bisulfites (SO_3_^2−^/HSO_3_^−^) at a molar ratio of 3:1 (Reaction 3). In the final stage, toxic sulfite is converted into harmless sulfate by the enzyme sulfite oxidase (SUOX) (Reaction 4) [11,12].

In cutaneous physiology, SO_2_ acts as a gasotransmitter and signals through several mechanisms: (1) the sGC/cGMP pathway, which is involved in cutaneous microcirculation [11]; (2) ion channels that ensure cellular excitability; (3) the cAMP/ protein kinase A (PKA) pathway, which facilitates smooth muscle relaxation; (4) S-sulfonation of proteins, which stabilizes the conformation and activity of enzymes and receptors; and (5) pathways involving NF-kB and mitogen-activated protein kinase (MAPK), which are critical in immune response, redox processes, inflammation, and oxidative stress [9,12,13].

The synthesis and degradation of SO_2_ in biological systems are governed by the dynamic balance between its production and catabolism. This equilibrium is influenced by the activity of key enzymes involved in its biosynthesis (e.g., CDO and AAT) and metabolism (e.g., SUOX), as well as by oxidative stress, inflammatory mediators, and interactions with other gasotransmitters within the cellular microenvironment [14].

CDO1 (E.C.1.13.11.20), the rate-limiting enzyme in cysteine catabolism, prevents the accumulation of cysteine in tissues, thereby avoiding toxicity associated with excess cysteine in the skin. It also indirectly modulates taurine and sulfate levels, which are important for hydration and maintaining skin barrier integrity, as well as the total glutathione (GSH) reserve, a key determinant of cellular antioxidant capacity [15]. Increased expression of CDO1 can lead to oxidative stress by reducing cysteine levels and decreasing GSH synthesis [15]. In dermatology, CDO has been studied in relation to the pathogenicity of dermatophyte infections [16], carcinomas [15], and Sezary syndrome [17]. Evaluating gene expression and epigenetic changes along with restoring CDO1 function, demonstrates clinical utility for diagnosis and the potential as a therapeutic target to overcome therapy resistance [18].

AAT (E.C.2.6.1.1.) is an aminotransferase that functions as a signaling molecule and plays a role in vital processes within skin tissues, such as collagen synthesis and the stability of mast cells [11]. The AAT/SO_2_ pathway has been observed in keratinocytes and immune cells within the skin. AAT self-regulates its synthesis through sulfenylation. AAT levels can vary in dermatoses or other conditions triggered by toxic exposure. AAT1 deficiency is associated with decreased SO_2_ production, increased mast cell degranulation, and occurrence of allergic reactions [19]. Targeting the AAT/SO_2_ pathway can disrupt the redox balance of the cell.

SUOX (EC 1.8.3.1) plays a crucial role in skin detoxification by converting sulfites into non-toxic sulfates. In pathophysiological conditions, when SUOX does not function properly, sulfites can accumulate, leading to oxidative stress and chronic inflammation. A decrease in SUOX activity, evaluated by serum levels of the enzyme and sulfites, is associated with an increase in oxidative stress, an alteration of antioxidant defense, and inflammatory responses [20,21].

In other words, the endogenous pathways responsible for SO_2_ production and AAT activity may be disrupted in patients with dermatological conditions, thereby contributing to disease onset and progression. A deficiency of endogenous SO_2_/AAT promotes the production of pro-inflammatory cytokines and suppresses protease-activated receptor 2 (PAR-2) expression in the skin through a non-histaminergic pathway [19].

## 3. The Role of SO_2_ in Cutaneous Homeostasis and Skin Pathology

Research on the role of endogenous SO_2_ in the skin is ongoing. While SO_2_ is beneficial in small amounts, an imbalance in its production can perpetuate cutaneous pathophysiological processes. At physiological concentrations, the endogenous SO_2_ produced by human skin cells—including vascular smooth muscle cells, endothelial cells, fibroblasts, dermal immune cell population, mast cells, keratinocytes, macrophages, and adipocytes—serves as a gasotransmitter with essential roles in skin physiology. It helps maintain cellular homeostasis by exhibiting various effects, including anti-inflammatory, antioxidant, vasodilatory, regenerative, antifibrotic, antiproliferative, immunosuppressive, and antiapoptotic properties [4,10,11,22]. Endogenous SO_2_ limits inflammation through several mechanisms: (1) reduction in the levels of pro-inflammatory interleukins (such as IL-1β, IL-6, and tumor necrosis factor alpha—TNF-α) through the inhibition of NF-κB; (2) suppression of the activity of metalloproteinases via their natural inhibitors (tissue inhibitors of metalloproteinase—TIMP); (3) stimulation of the production of endogenous antioxidants that neutralize reactive oxygen species via the Nrf2 pathway; (4) promotion of the transition to the tissue repair phase through the interaction with signaling pathways (including activation of MAPK and inactivation of p38 and Jun N-terminal kinase—JNK); (5) exertion of local immunosuppressive effects by regulating ion channels and stabilizing mast cells, thereby blocking overactive immune responses [22,23].

Endogenous SO_2_ plays a vital role in maintaining the skin’s protective barrier due to its antioxidant properties. The effect is mediated through the regulation of GSH, increasing the production of antioxidant enzymes, and inhibiting pro-oxidative systems. These beneficial effects are enabled through the activation of the Nrf2 pathway and the inactivation of NADPH oxidase [9,24].

At physiological concentrations, SO_2_ promotes the relaxation of smooth muscles in blood vessel walls, which enhances microcirculation in the skin and improves the transport of nutrients and oxygen to tissues. This regulation occurs through the modulation of ion channels, activation of cyclic GMP (cGMP), and interaction with nitric oxide (NO). Additionally, in the vascular system, SO_2_ inhibits the migration and proliferation of smooth muscle cells, functions that are crucial for maintaining skin homeostasis [9,11].

Endogenous SO_2_ also modulates the skin’s barrier function and promotes skin regeneration by reducing fibrosis and modulating cell remodeling. It regulates the proliferation and differentiation of keratinocytes and fibroblasts, maintaining the balance between collagen and elastin synthesis and degradation. The mechanisms by which SO_2_ preserves the structural integrity of the skin are complex and involve: (1) sulfenylation of cysteine residues in proteins (such as Smad3, NF-κB p65, or AAT); (2) modulation of NF-κB pathways; (3) modulation of Nrf2/HO-1 and ERK1/2/p38 signaling pathways; (4) regulation of amphiregulin and filaggrin levels [22,25].

The immunosuppressive effects of endogenous SO_2_ in the dermis are supported by inhibiting mast cell degranulation, preventing the activation and infiltration of T cells and neutrophils into the dermis, and downregulating the transcription of pro-inflammatory genes in skin cells through the modulation of the NF-κB pathway. This process involves inhibition of the phosphorylation of the p38 and JNK signaling pathways, as well as interaction with H_2_S [22,26].

Endogenous SO_2_ influences mitochondrial function and can protect cells against apoptosis under hypoxic conditions. The gas prevents uncontrolled cell death in the skin by reducing inflammation (via NF-κB), activating cell survival pathways (such as phosphoinositide 3-kinase/protein kinase B—PI3K/Akt, MAPK, and p38), and increasing the Bcl-2/Bax ratio, which decreases pro-apoptotic Bax expression and increases anti-apoptotic Bcl-2 [10,12].

SO_2_ imbalance, whether due to excess production or deficiency, is a contributing factor in the development of various chronic skin conditions. Endogenous SO_2_ deficiency can lead to several issues: (1) It accelerates the degradation of skin support fibers, which is associated with premature skin aging [10]; (2) It promotes endothelial dysfunction, resulting in vasculitic lesions or altered wound healing [27]; (3) It supports the hyperproliferation of keratinocytes, the hallmark feature of psoriasis [10]; (4) It exacerbates the inflammatory processes accompanying atopic dermatitis and eczema; (5) It induces mast cell hyperactivation, leading to the rapid release of pro-inflammatory mediators (like histamine, cytokines, and leukotrienes) that worsen neurogenic inflammation and itching in conditions such as urticaria, atopic dermatitis, and psoriasis [4]; (6) It causes the accumulation of dysfunctional collagen or abnormal remodeling of connective tissue, features characteristic of conditions like collagenosis, skin aging, cutaneous sclerosis, and fibrotic diseases [28,29]; (7) It dysregulates cellular mechanisms controlling apoptosis and angiogenesis, which are critical in carcinogenesis (melanoma, lymphoma, carcinomas) [12,30]; (8) It leads to dysbiosis of the skin microbiome, worsening seborrheic dermatitis, atopic dermatitis, acne vulgaris and psoriasis [4,31].

When the production of endogenous SO_2_ and its derivatives (sulfite/bisulfite) exceed the cell’s capacity to detoxify, it becomes harmful to cells. The effects at the cellular level include: (1) mitochondrial dysfunction and the alteration of protein structures, resulting in the breakdown of polypeptide chains [10]; (2) oxidative damage due to increased oxidative stress; (3) the triggering of apoptosis and inflammation through the massive release of pro-inflammatory cytokines and activation of the NF-kB pathway [9]; (4) alterations in skin integrity by disrupting the balance of cell synthesis and differentiation, as well as the degradation of keratins, collagen, and elastin due to the reducing effects of sulfites [9,25].

It is widely accepted that SO_2_ plays a significant role in maintaining intracellular redox homeostasis; however, some aspects of its redox biology remain unclear. For instance, it is not fully understood which are the main target molecules that SO_2_ and its derivatives bind to. Increased concentrations of SO_2_ lead to the production of reactive oxygen species, which disrupt redox homeostasis and cause damage to proteins, lipids, and nucleic acids [9]. Sulfur-derived free radicals, such as SO_3_•^−^ and SO_4_•^−^, produced during the process of autooxidation of sulfite (SO_3_^2−^), can damage DNA [32]. Furthermore, the exposure to SO_2_ results in lipid peroxidation, which is indicated by elevated levels of thiobarbituric acid reactive substances (TBARS) observed in subjects exposed to high concentrations of SO_2_. This exposure also leads to a decrease in antioxidant levels, affecting the activity of antioxidant enzymes such as superoxide dismutase and glutathione peroxidase [33]. Additionally, bisulfite can interact with metalloproteins, exemplified by sulfite oxidase, as well as with electrophilic compounds, resulting in modulation of their activities. Animal studies have demonstrated these effects across multiple organs, including the lungs, heart, liver, stomach, intestines, and spleen, indicating that SO_2_ acts as an oxidizing agent with systemic effects at elevated concentrations [32,33].

Understanding the effects and mechanisms of exogenous SO_2_ on skin cells is crucial, especially given the growing concerns about air pollution and its impact on skin health. SO_2_ is a significant pollutant; toxicological studies reveal that its derivatives (can penetrate the skin layers, causing cellular damage through oxidative and inflammatory pathways. Recent epidemiological studies indicate that exposure to air pollution (including particulate matter of various sizes, polycyclic aromatic hydrocarbons, gaseous components like SO_2_, and volatile organic compounds) exacerbates numerous inflammatory skin conditions, such as atopic dermatitis, eczema, pruritus, psoriasis, as well as allergic or hypersensitivity reactions (e.g., urticaria) and autoimmune skin diseases (e.g., cutaneous lupus erythematosus, scleroderma). Additionally, air pollution contributes to accelerated skin aging, hair loss, or skin tumor development (melanoma, basal and squamous cell carcinomas) [6,34,35].

The pathogenic mechanisms by which air pollutants affect inflammatory skin diseases primarily involve the skin microbiome, the aryl hydrocarbon receptor (AhR) pathway, oxidative stress, and the inflammasome [36]. Current research indicates that environmental air pollution can exacerbate acne by altering the skin’s lipid composition and provoking inflammation [3].

Epidemiological data have established a link between pollutants and skin tumors, likely due to the activation of keratinocytes and melanocytes. Atmospheric pollutants contribute to the development of cutaneous tumors through multiple mechanisms, including the formation of DNA adducts via reactive intermediates such as epoxides and diols, induction of oxidative stress and genotoxicity, and activation of the AhR pathway [36].

Furthermore, studies have indicated that animals lacking Langerhans cells demonstrate a lower susceptibility to cancer. Langerhans cells play a role in the metabolic conversion of pollutants into pro-oncogenic intermediates, increasing mutagenesis rates and causing DNA damage in the epidermis, which subsequently contributes to the development of squamous cell carcinomas [34,37]. SO_2_, as a co-pollutant, can induce epigenetic remodeling. These alterations occur through DNA methylation affecting antioxidant and immunoregulatory genes and the acetylation of histones at inflammatory loci [3]. These events may help explain why there is an increased risk of disease long after the initial exposure.

In conclusion, endogenous SO_2_ serves as an essential signaling molecule that significantly impacts skin homeostasis and metabolic balance. Endogenous SO_2_ exhibits a dual role, functioning as a physiological regulator under normal conditions while acting as a potentially deleterious agent in pathological states. These findings are valuable for developing new therapeutic agents, such as SO_2_ donors or inhibitors, for the treatment of dermatological diseases including melanoma [25,28,38].

## 4. SO_2_ Interaction with Skin Cells Under Physiological and Pathological Conditions

Many physiological processes in human skin are mediated by SO_2_, a gaseous signaling molecule. Almost every type of skin cell can produce SO_2_. The interaction of SO_2_ with skin cells is complex, exhibiting distinct physiological and pathophysiological roles. Understanding the effects and mechanisms of SO_2_ is crucial in modern dermatology and cell biology. This encompasses both its internal production, which may provide protective and therapeutic benefits, and its external exposure, which can have toxic and irritating effects. This understanding shifts the perception of SO_2_ from merely being an air pollutant to being recognized as an important modulator in dermatological and immunological responses within both healthy and affected skin [36].

### 4.1. The Role of SO_2_ in Modulating Mast Cell Activity

Mast cells are abundantly distributed throughout the body and frequently interact with the external environment. Cutaneous mast cells are located in the dermis near blood vessels, nerve endings, and hair follicles, and they play a crucial role in immune protection, maintaining the skin’s barrier, facilitating neuroimmune communication, and mediating allergic reactions [22]. Endogenous SO_2_, together with stem cell factor (SCF)/KIT receptor signaling—associated with activation of ERK, PI3K/AKT, and STAT5 pathways—as well as IL-33/ST2 signaling—linked to activation of p38 and JNK pathways [39]—collectively orchestrates mast cell survival and functional activity [22].

At physiological concentrations, endogenously generated SO_2_ acts as a mast cell stabilizer, a phenomenon demonstrated both in vivo and in vitro [22]. Recent research has identified two primary molecular mechanisms through which endogenous SO_2_ stabilizes mast cells: (1) Activation of the SO_2_-modulated cAMP signaling pathway: increased intracellular cAMP levels—via stimulation of adenylate cyclase and inhibition of phosphodiesterase—prevent mast cell degranulation and the release of inflammatory mediators [13]; (2) SO_2_-mediated redox alteration: a specific post-translational mechanism by which SO_2_ inhibits IgE- or hypoxia-induced mast cell activation involves the sulfenylation of galectin-9 at cysteine residue 74. Galectin-9 is a key regulatory molecule in the control of immune cell activity [22].

The level of SO_2_ and sulfenylated galectin-9 at the Cys74 site may serve as a regulatory switch for degranulation and activation of mast cells in both physiological and pathological conditions. Mast cell degranulation is a key pathological process in various conditions, including cutaneous mastocytosis, mast cell activation syndrome, angioedema, and chronic dermatoses such as atopic eczema, contact dermatitis, psoriasis, prurigo, and rosacea [22,39].

Mast cell activation generally follows two pathways: (1) the classical IgE-mediated pathway, which occurs when the high-affinity Fc receptor for IgE (FcεRI) binds to mast cells, and (2) the non-IgE-mediated pathway, which induces mast cell degranulation through exposure to stem cell factors, endothelin-1, and neuropeptide E. The downregulation of the SO_2_/AAT1 pathway may play a significant role in the pathogenesis of diseases related to mast cell activation. The endogenous SO_2_/AAT metabolic pathway, present in myeloid cells, regulates mast cell degranulation induced by allergies or hypoxia/inflammation by activating cyclic adenosine monophosphate/protein kinase A/phosphodiesterases (cAMP/PKA/PDE) and inhibiting Raf/MEK/ERK [22]. In conclusion, endogenous SO_2_ acts as a regulator that reduces the severity of skin allergic reactions by blocking histamine release from mast cells (Table 1).

Downregulation of the endogenous SO_2_/AAT pathway in hypoxic microenvironments, often associated with inflammation, may promote mast cell degranulation and increase sensitivity to IgE-mediated degranulation. Mast cell activation in these hypoxic environments enhances angiogenesis and vascular remodeling by increasing matrix metalloproteinase activity [22].

External exposure to sulfur compounds (e.g., SO_2_ and sulfites) can trigger or exacerbate skin reactions such as pruritus and urticaria. Chronic exposure leads to the release of inflammatory mediators, worsening the allergic immune response. Sodium sulfite can induce pyroptosis, which is an inflammatory form of programmed cell death characterized by the lysis of the plasma membrane. Additionally, it can lead to non-IgE degranulation, mast cell sensitization, increased intracellular oxidative stress, and the amplification of NLRP3, caspase-1, N-terminal D gasdermin, IL-1β, and IL-18 expression [40]. However, supplementation with SO_2_ can reverse these processes, thereby protecting tissues from inflammation-induced vascular remodeling [41].

### 4.2. Impact of SO_2_ on Skin Pigmentation

Harmful compounds in the environment, including SO_2,_ act as external stressors that penetrate the skin, directly affecting the proper cell division and function of melanocytes. External SO_2_ acts as an oxidant and irritant, inducing oxidative stress and chronic inflammation in the skin. These biochemical processes trigger or exacerbate melanogenesis as a defensive response, leading to hyperpigmentation disorders such as melasma, senile lentigo, and diffuse facial pigmentation [2,42,43]. Skin pigmentation plays a significant role in protecting against external stress [44].

Currently, there is no definitive consensus in the literature on the correlation between air pollution and the incidence rate of cutaneous melanoma. Mendelian randomization analysis has shown no statistically significant association between air pollution and the risk of developing cutaneous melanoma. Furthermore, substantial evidence does not support a causal relationship between air pollution and melanoma risk within European populations [45].

Research indicates that biochemical processes related to endogenous SO_2_ are linked to melanin production. Melanin, a natural pigment, is produced in specialized organelles called melanosomes. Melanogenesis is a complex process involving multiple molecules and signaling pathways [44]. Among these factors, endogenous SO_2_ may influence intracellular signaling pathways that regulate melanogenesis (ERK1/2 and p38 signaling pathways) [42,46]. SO_2_ helps maintain melanocyte homeostasis under physiological conditions through antioxidant mechanisms, modulation of apoptosis, and balancing eumelanin and pheomelanin in the skin (Table 2) [44].

SO_2_ acts as an inhibitor of melanogenesis due to its strong potential to reduce and inhibit the activity of tyrosinase and peroxidase [47]. Dysregulation of SO_2_ levels can contribute to pigmentary disorders such as vitiligo or melasma. Tyrosinase plays a crucial role in controlling both depigmentation and hyperpigmentation [18]. While SO_2_ can have signaling roles in small quantities, excess SO_2_ acts as a pro-oxidant, inducing dysfunction in melanin production, which may lead to either hypopigmentation or hyperpigmentation.

Given that oxidative stress triggers melanogenesis, the reduction in reactive oxygen species by endogenous SO_2_ may help limit hyperpigmentation [48]. Excess SO_2_ exposure has been reported to upregulate reactive oxygen species, disrupt redox homeostasis, deplete GSH, and inactivate biomacromolecules such as proteins, lipids, and DNA. In addition, the reactive oxygen species generated during homocysteine oxidation may trigger melanocyte apoptosis [47].

### 4.3. SO_2_—As a Regulator of Microcirculation

In the skin, endogenous SO_2_ serves as a regulator of microcirculation (Table 3). At physiological concentrations, SO_2_ helps maintain normal vascular structure by suppressing the excessive proliferation of vascular smooth muscle cells and collagen deposition [49]. It regulates vascular function through several pathways: (1) cAMP/PKA pathway: SO_2_ increases cAMP levels, which activate protein kinase A (PKA). This process blocks c-Raf through phosphorylation at the inhibitory site Ser259, leading to the inactivation of the ERK/MAPK pathway, thereby inhibiting the proliferation of smooth muscle cells [3]; (2) TGF-β1/Smad2/3 Pathway: SO_2_ inhibits this pathway, resulting in reduced collagen synthesis and collagen degradation [23]; (3) antioxidant mechanisms: SO_2_ reduces oxidative stress in vascular tissues, prevents the excessive production of reactive oxygen species, and stimulates antioxidant defenses [3].

While the endogenous SO_2_/AAT pathway may exert protective effects on the vascular endothelium, reduced AAT activity and increased concentrations of exogenous SO_2_ can lead to oxidative damage, impairing Nrf2 signaling. In models of oxidative damage caused by SO_2_, AAT downregulation is associated with reduced transcription and oxidative modification of cysteine residues with catalytic functions from AAT, resulting in persistent vascular lesions [3]. Ion channels (such as L-type calcium channels and K/ATP channels), as well as cGMP and cAMP pathways, are involved in the vasorelaxant effects of SO_2_. Additionally, the MAPK pathway is important for vascular remodeling and the proliferation of vascular smooth muscle cells [23]. SO_2_ supplementation may favorably influence arterial or pulmonary hypertension. NO signaling pathway is also involved in this process, potentiating the vasodilator effect of SO_2_, the inhibition of nitric oxide synthase (NOS) exerting a negative effect [13,41].

### 4.4. SO_2_ Interface with Cutaneous Fibroblasts

Endogenous SO_2_ mediates the fibrosis process by inhibiting excessive fibroblast proliferation and suppressing collagen remodeling. The mechanisms underlying the inhibition of skin fibrosis by SO_2_ are associated with its antioxidant and anti-inflammatory effects, as well as its ability to inhibit cell proliferation [25,29]. It is estimated that endogenous SO_2_ can enhance collagen degradation by regulating the balance between matrix metalloproteinases (MMPs) and tissue metalloproteinase inhibitors, which play a role in stabilizing vascular architecture and connective tissue structure [50].

Connective tissue degradation is mediated by MMPs [51]. Collagen degradation is initiated by collagenase (MMP-1) and is further processed by stromelysins (MMP-3) and gelatinases (MMP-2 and MMP-9), resulting in skin aging [43]. Dysfunction in the SO_2_/AAT pathway and decreased production of endogenous SO_2_ may be linked to the development of fibrotic processes and abnormal collagen accumulation and dysregulation of MMPs/TIMP balance. Thus, endogenous SO_2_ exerts antifibrotic effects through the regulation of tissue remodeling mechanisms [29].

At the cutaneous level, aberrant signaling involving TGF-β, Wnt/β-catenin, Notch, and JAK/STAT6 pathways, together with dysregulation of the extracellular matrix (ECM), IL-4/IL-13 interleukins, and epithelial–mesenchymal interactions, contribute to fibrogenesis (fibroblast activation and extracellular matrix accumulation) [25].

In addition, air pollutants, including SO_2_, contribute to oxidative stress, mitochondrial damage, and accelerated skin aging, leading to cutaneous fibrogenesis [25]. In response to injury or stress, fibroblasts undergo adaptive proliferation, which is associated with increased collagen synthesis. However, the excessive accumulation of collagen, resulting from overproliferation of fibroblasts, can lead to fibrosis and vascular dysfunction, both of which are involved in the pathogenesis of skin conditions (see Table 4). SO_2_ can inhibit fibroblast proliferation through post-translational mechanisms, including the sulfenylation of ERK1/2, NF-κB p65 (at Cys38), and Smad3 (at Cys65). Additionally, it inhibits the phosphorylation of ERK1/2 and Smad3, attenuates inflammatory responses, and regulates collagen synthesis [50]. Overexpression of the enzymes AAT1 and AAT2 negatively regulates collagen synthesis, while inhibition of AAT1 or AAT2 can promote excessive collagen accumulation in vascular smooth muscle cells [29].

### 4.5. The Interaction Between SO_2_ and Keratinocytes

Keratinocytes are essential for maintaining the skin’s protective barrier and facilitating wound healing. This physiological process involves several cellular events, including the proliferation, differentiation, and migration of keratinocytes. They secrete cytokines, chemokines, and growth factors that regulate the skin’s immune response and help maintain tissue homeostasis [52]. Both SO_2_ and supersulfides play key roles in keratinocyte migration and support skin wound healing [53]. At physiological concentrations, endogenous SO_2_ has a cytoprotective effect on keratinocytes by maintaining redox balance and regulating glutathione levels. It also influences cell differentiation, modulates inflammation by inhibiting pro-inflammatory cytokine pathways, and regulates cell proliferation (Table 5). These effects may be mediated by activating the NF-κB pathway and inhibiting the ERK1/2 and p38 pathways [24]. A deficiency in the endogenous SO_2_/AAT pathway can lead to cellular senescence, which is associated with the nuclear translocation of STAT3 and a reduction in STAT3 sulfenylation at Cys 259. Post-translational changes in cysteine residues can significantly affect protein function, activity, adhesion, migration, conformation, localization, and interactions with other molecules [54].

The external introduction of SO_2_ or its derivatives into keratinocyte cultures can disrupt skin homeostasis. This disruption is characterized by increased oxidative stress, as evidenced by elevated levels of radical oxygen species, malondialdehyde, tumor necrosis factor alpha (TNF-α), interleukin-1 beta (IL-1β), Nrf2, heme oxygenase-1 (HO-1), and phosphorylated p65 (p-p65). At the same time, the cells exhibit reduced antioxidant capacity, reflected by decreased levels of superoxide dismutase (SOD), IκB, phosphorylated ERK1/2 (p-ERK1/2)/ERK1/2, and phosphorylated p38 (p-p38). This disruption activates inflammatory signaling and leads to keratolytic effects, including the destruction of disulfide bonds between keratinocytes and the disintegration of the stratum corneum. SO_2_ can inhibit the proliferation of human skin keratinocytes and induces oxidative stress and inflammation by activating the NF-κB pathway and inactivating ERK1/2 and p38 [24,28].

Increasing the expression of AAT2 and SO_2_ may be a promising strategy for developing novel anti-aging therapies [27]. Gas therapy could serve as a modern regenerative therapeutic solution for wound healing [55].

### 4.6. The Role of SO_2_ in Macrophage Polarization

The skin, as the largest immune organ in the human body, provides an interesting model for studying macrophage polarization in inflammatory skin diseases and tumors, such as atopic dermatitis, psoriasis, lichen planus, systemic sclerosis, systemic lupus erythematosus, rosacea, bullous pemphigoid, melanoma, and cutaneous T-cell lymphoma [56,57]. Macrophages are vital immune cells that help control immunological and inflammatory responses, exhibiting a dual role in the pathology of inflammatory diseases. They can function both as promoters of inflammation and as repair agents for damaged tissues [58,59].

Under physiological conditions, endogenous SO_2_ plays a crucial role in regulating macrophage polarization, particularly by inhibiting the pro-inflammatory M1 phenotype and mitigating inflammation [60]. Macrophage-derived SO_2_ is a significant regulator of macrophage activation [61]. A deficiency of endogenous SO_2_, caused by the downregulation of SO_2_/AAT2 in macrophages, can trigger spontaneous inflammation, indicated by elevated levels of TNF-α and IL-6, as well as increased macrophage chemotaxis [61]. In contrast, exogenous SO_2_ functions as a stressor that impairs macrophage activity, causing them to shift from defensive cells to contributors of chronic inflammation. By modulating levels of SO_2_, the polarization of M1 macrophages is controlled through the sulfenylation of prolyl hydroxylase at Cys260 (Table 6) [60].

**Figure 2:**
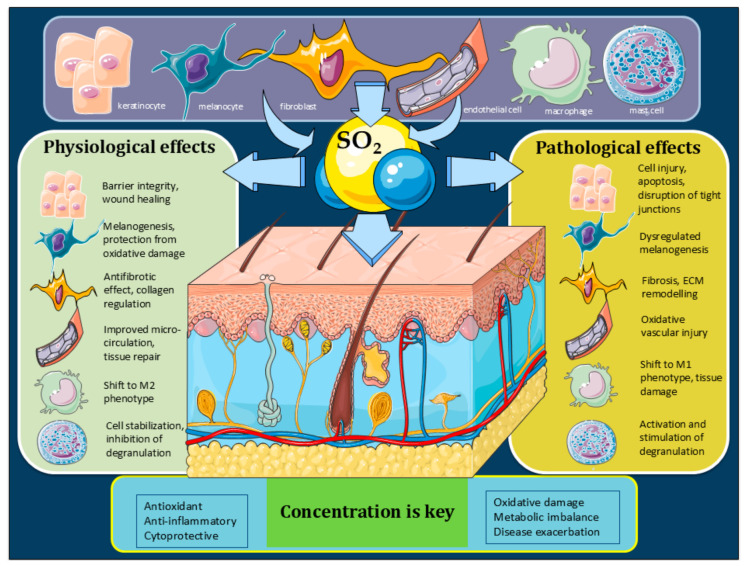
The dual role of SO_2_.

In summary, SO_2_ has a dual role on skin cells, depending on its concentration (Figure 2).

### 4.7. The Potential Roles of SO_2_ in the Skin Microbiome

Maintaining a balance between the host and commensal microorganisms is essential for skin health. Air pollutants, including SO_2_, as well as endogenous SO_2_ deficiency, have a significant negative impact on skin surface biodiversity, structure, function, and the modulation of the skin microbiome in both health and disease (Table 7). Changes in SO_2_ levels and endogenous sulfur species are associated with abnormal functioning of the skin barrier, leading to imbalances in microbial communities. This can contribute to the onset and prevalence of inflammatory and infectious skin diseases, accelerate skin aging, and influence the progression of skin cancers. Distinct dysbiotic patterns have been identified in various dermatological diseases, including acne, atopic dermatitis, chronic wounds, psoriasis, seborrheic dermatitis, actinic keratosis, squamous cell carcinoma, cutaneous melanoma, basal cell carcinoma, cutaneous T-cell lymphoma, alopecia areata, vitiligo, and rosacea [31,62]. An integrative analysis of the skin microbiome—which includes Gram-positive and Gram-negative bacteria, fungi, and parasites—along with the profile of skin surface metabolites (derived from both microbes and host), can shed light on host-microbe interactions and help understand the pathogenesis of skin diseases linked to cutaneous dysbiosis [63].

In dermatology, CDO has been investigated in relation to the pathogenicity of dermatophytes. These fungi produce sulfites that can damage keratin found in the skin, nails, or hair of infected individuals [16]. Endogenous sulfur compounds, including SO_2_, are involved in signaling pathways such as Nrf2, NF-κB, and MAPK/AP-1, which mediate the pathogenesis of inflammatory skin diseases [31].

Changes in the microbiome can also affect the metabolism of sulfur-containing amino acids on the skin’s surface. For example, the metabolism of sulfur, cysteine, and methionine is significantly increased in psoriasis lesions. This increase can be explained by the fact that the abnormal proliferation of keratinocytes and inflammatory responses in psoriasis increase the demand for amino acids [63]. The skin’s microbiome contributes to the overall redox environment, influencing the production of sulfur compounds. Compounds containing sulfur play a crucial role in the microbiome by providing protective and anti-inflammatory effects. They also help prevent the adhesion of pathogenic microorganisms to the epithelial surface. However, when an imbalance occurs and these compounds accumulate in large quantities, the microbiome microorganisms can disrupt systemic redox homeostasis and increase oxygen utilization [64].

Microorganisms interact with these sulfur compounds to form an antioxidant environment. Changes in the microbiome can decrease sebaceous gland activity, resulting in dry skin and lower levels of beneficial sulfur metabolites. This creates conditions that favor pathogen colonization [65].

## 5. SO_2_—As a Therapeutic Agent

Recently, gas therapy has emerged as a promising alternative for treating tumors, healing skin wounds, antimicrobial applications, and combating fibrosis [9,66,67]. Various studies are exploring the role of reactive sulfur species (RSS), such as H_2_S, SO_2_, polysulfides (RSSnR), and hydropersulfides (RSSnH), in numerous biological processes aimed at restoring tissue homeostasis and biological functions [38,67,68]. The primary mechanisms of action for these compounds involve S-persulfuration of proteins (-SSH) and S-sulfenylation of proteins (-SOH) [22,68].

RSS donors have demonstrated anti-inflammatory effects by suppressing NF-κB signaling, modulating the NLRP3 inflammasome, and blocking the JAK/STAT pathways [10,68]. However, challenges such as insufficient or uncontrolled gas administration, unclear therapeutic mechanisms, and gaps in the dose–response relationship limit their clinical application. Research on the therapeutic potential of SO_2_ indicates that modulating its production through exogenous SO_2_ donors, genetic manipulation of biosynthetic enzymes (CDO, AAT, SUOX), or lifestyle interventions could yield promising results in treating allergic and inflammatory skin conditions. Nonetheless, many questions remain that require further investigation.

## 6. Conclusions

The current analysis of the effects and mechanisms mediated by SO_2_ in healthy and damaged skin suggests that this recently validated gasotransmitter could play a crucial role in skin health. This analysis, unique in the literature, provides a narrative synthesis of the current evidence related to SO_2_ as a gasotransmitter in both healthy and affected skin.

SO_2_ is recognized as an essential regulator of redox homeostasis, anti-inflammatory signaling, and mitochondrial function. It influences skin immune responses, microcirculation, pigmentation, normal barrier function, and the balance between host and commensal microorganisms. Additionally, it promotes the repair of skin tissues and reduces inflammatory lesions, highlighting its significance in dermatological diseases.

SO_2_ acts as a signaling messenger, playing a vital role in skin homeostasis, physiology, and pathology. At physiological concentrations, SO_2_ is cytoprotective; however, at abnormal concentrations, it can be toxic. This duality—serving as both a toxicological agent and a physiological modulator—underscores the complexity of its biological effects and emphasizes the need for further research.

On the other hand, the effects attributed to SO_2_ may reflect the combined impact of several pollutants on the skin or the interaction of multiple endogenous signaling molecules within skin tissues. These factors complicate the identification of SO_2_ specific contributions, making definitive causal relationships uncertain. Future investigations should also consider the broader systemic impacts of gasotransmitters on skin physiology and pathology.

Because skin pathology is closely interconnected with metabolic and immune processes, the relationship between SO_2_ and the skin should be interpreted within a holistic and integrative framework rather than through strictly deterministic conclusions. Assessing the relationship between SO_2_ and health outcomes requires a comprehensive approach that accounts for factors such as climate change, air pollution, and various physiological and pathological conditions.

## Figures and Tables

**Table 1:** Summary of the Mechanisms Mediated by SO_2_ and the Effects on Mast Cells in Allergic Skin Responses.

SO_2_ Source	Effect	Biological Context	Mechanism
Endogenous (active AAT1)	Protective	Mast cell stabilization	cAMP signaling and protein sulfenylation
Endogenous (reduced AAT1 activity)	Allergic response	Activation of mast cell degranulation	cAMP/PKA/PDE signaling and Raf/MEK/ERK pathways
Exogenous (pollution, food additives)	Irritant	Sensitization and intolerance	Inflammation, oxidative stress, and pyroptosis
SO_2_ supplementation	Therapeutic	Tissue repair	Anti-inflammatory effects and desensitization

SO_2_—sulfur dioxide; AAT1—aspartate aminotransferase 1; cAMP—cyclic adenosine monophosphate; PKA—protein kinase A; PDE—phosphodiesterase; Raf—rapidly accelerated fibrosarcoma kinase; MEK—mitogen-activated protein kinase kinase; ERK—extracellular signal-regulated kinase.

**Table 2:** Summary of the Effects and Mechanisms Mediated by the SO_2_–Melanocyte Interaction.

SO_2_ Source	Effect	Biological Context	Mechanism
Endogenous (active AAT1)	Stabilizing	Effective inhibition of tyrosinase activity	Modulation of ERK1/2 and p38 signaling pathways
Endogenous (reduced AAT1 activity)	Activating	Enhanced melanogenesis	Disruption of the pheomelanin/eumelanin ratio
Exogenous	Pro-oxidant	Uncontrolled pigmentation	Induction of oxidative damage
SO_2_ supplementation	Beneficial	Melanoma treatment	Induction of oxidative stress–mediated apoptosis in malignant cells

SO_2_—sulfur dioxide; AAT1—aspartate aminotransferase 1; ERK1/2—extracellular signal-regulated kinases 1 and 2; p38—p38 mitogen-activated protein kinase.

**Table 3:** Summary of the Effects and Mechanisms Mediated by the Impact of SO_2_ on Vascular Skin Cells.

SO_2_ Source	Effect	Biological Context	Mechanism
Endogenous (active AAT1)	Protective	Maintenance of vascular integrity	Activation of cAMP/PKA and cGMP/PKG signaling pathways
Endogenous (reduced AAT1 activity)	Detrimental	Oxidative vascular injury	Involvement of the Nrf2 signaling pathway
Exogenous (pollution, additives)	Cytotoxic	Collagen deposits in tissues	Inflammation and oxidative stress
SO_2_ supplementation	Beneficial	Tissue regeneration	ERK/MAPK pathways; eNOS signaling

SO_2_—sulfur dioxide; AAT1—aspartate aminotransferase 1; cAMP—cyclic adenosine monophosphate; PKA—protein kinase A; cGMP—cyclic guanosine monophosphate; PKG—protein kinase G; Nrf2—nuclear factor erythroid 2–related factor 2; ERK—extracellular signal-regulated kinase; MAPK—mitogen-activated protein kinase; VEGF—vascular endothelial growth factor; eNOS—endothelial nitric oxide synthase.

**Table 4:** Summary of the Effects and Mechanisms Mediated by the Impact of SO_2_ on Cutaneous Fibroblasts.

SO_2_ Source	Effect	Biological Context	Mechanism
Endogenous (active AAT1)	Protective	Downregulation of fibroblast proliferation	Mechanisms involving tissue homeostasis
Endogenous (reduced AAT1 activity)	Fibrogenic	Abnormal collagen accumulation	MMPs/TIMPs
Exogenous (pollution, additives)	Proliferative	Skin fibrosis	Mitochondrial dysfunction and oxidative stress
SO_2_ supplementation	Anti-fibrotic	Inhibition of fibroblast proliferation	SO_2_-mediated protein sulfenylation

SO_2_—sulfur dioxide; AAT1—aspartate aminotransferase 1; MMPs—matrix metalloproteinases; TIMPs—tissue inhibitors of metalloproteinases.

**Table 5:** Summary of the Effects and Mechanisms Mediated by the Impact of SO_2_ on Keratinocytes.

SO_2_ Source	Effect	Biological Context	Mechanism
Endogenous (active AAT1)	Cytoprotective	Maintenance of keratinocyte integrity	Modulation of NF-κB, ERK/p38 MAPK, and Nrf2 signaling pathways
Endogenous (reduced AAT1 activity)	Increased susceptibility to oxidative stress	Inhibition of keratinocyte migration	Impaired wound healing and regeneration
Exogenous (pollution, additives)	Cytotoxic	Keratinocyte dysfunction	Inflammation and oxidative stress
SO_2_ supplementation	Protective	Reduction in cellular senescence	Upregulation of AAT/SO_2_ pathway activity

SO_2_—sulfur dioxide; AAT1—aspartate aminotransferase 1; NF-κB—nuclear factor kappa B; ERK—extracellular signal-regulated kinase; p38 MAPK—p38 mitogen-activated protein kinase; Nrf2—nuclear factor erythroid 2–related factor 2; AAT—aspartate aminotransferase.

**Table 6:** Summary of Effects and Mechanisms Mediated by the Impact of SO_2_ on Macrophages.

SO_2_ Source	Effect	Biological Context	Mechanism
Endogenous (active AAT1)	Cytoprotective	Regulation of macrophage activation	Inhibition of M1 phenotype polarization
Endogenous (reduced AAT1 activity)	Pro-inflammatory	Spontaneous inflammatory state	Reduced AAT2 levels
Exogenous (pollution, additives)	Stress-inducing	Macrophage dysfunction	Triggering of inflammatory responses
SO_2_ supplementation	Beneficial	Control of macrophage polarization (M1)	Sulfenylation of prolyl hydroxylase 2

SO_2_—sulfur dioxide; AAT1—aspartate aminotransferase 1; AAT2—aspartate aminotransferase 2; M1—classically activated (pro-inflammatory) macrophage phenotype.

**Table 7:** Summary of the Effects and Mechanisms Mediated by the Impact of SO_2_ on the Skin Microbiome.

SO_2_ Source	Effect	Biological Context	Mechanism
***Endogenous (active AAT1)***	Immunomodulatory	Host–microbiome modulation	Immune mechanisms involving host microbiome interaction
***Endogenous (reduced AAT1 activity)***	Detrimental	Dysbiosis	Reduced tissue repair capacity
***Exogenous (pollution, additives)***	Toxic	Microbial imbalance	Inflammation and oxidative damage
***SO_2_ supplementation***	Beneficial	Restoration of microbial communities	Normalization of SO_2_ levels

SO_2_—sulfur dioxide; AAT1—aspartate aminotransferase 1.

## Data Availability

No new data were created or analyzed in this study.
